# The need for genetic variant naming standards in published abstracts of human genetic association studies

**DOI:** 10.1186/1756-0500-2-56

**Published:** 2009-04-14

**Authors:** Wei Yu, Renée Ned, Anja Wulf, Tiebin Liu, Muin J Khoury, Marta Gwinn

**Affiliations:** 1 Office of Public Health Genomics, Centers for Disease Control and Prevention, Atlanta, Georgia 30341, USA

## Abstract

We analyzed the use of RefSNP (rs) numbers to identify genetic variants in abstracts of human genetic association studies published from 2001 through 2007. The proportion of abstracts reporting rs numbers increased rapidly but was still only 15% in 2007. We developed a web-based tool called Variant Name Mapper to assist in mapping historical genetic variant names to rs numbers. The consistent use of rs numbers in abstracts that report genetic associations would enhance knowledge synthesis and translation in this field.

## Discussion

By identifying millions of single nucleotide polymorphisms (SNPs), high-throughput genotyping technology has dramatically boosted the yield of genetic association studies [1]. Translating these data into useful health information depends on systematic review and knowledge synthesis [2]. However, the inconsistent description of key data elements – such as gene names, gene variant names, and measures of association – makes retrieval of published information challenging. Names for genes and polymorphisms are particularly problematic because historical or common names have often been used instead of standard nomenclature [3,4], particularly in candidate gene association studies.

The National Library of Medicine (NLM) provides free access via PubMed [5] to the most comprehensive repository of biomedical literature abstracts in the world. Thus, the efficiency and sensitivity of scientific literature searches, as well as the robustness of computerized processes for data and text mining, depend closely on the way that information is presented in PubMed abstracts. By using standard names for genes and genetic variants in published abstracts, authors can increase the accessibility, utility, and influence of their findings.

The Human Genome Epidemiology (HuGE) Navigator is an integrated and searchable knowledge base of human genetic associations that have been extracted from PubMed weekly since 2001 by a combination of automatic and manual processes [6]. The curator indexes each new abstract with the relevant HUGO gene symbol(s) [4], so that users can perform gene-specific queries that can also accommodate gene aliases or protein names. For systematic review and synthesis of gene-disease associations, more specific data – at the level of the genetic variant – are required. The National Center for Biotechnology Information (NCBI) has developed the SNP database (dbSNP) [7] as a central repository for SNPs and other genetic variants, each of which is identified by a unique reference cluster number (rs number).

**Figure 1:**
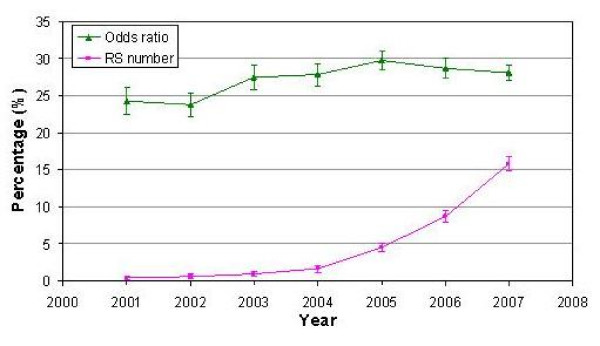
**Trends in the percentage of abstracts reporting odds ratios and rs numbers for gene variants, HuGE Navigator database, 2001–2007**.

We examined with the HuGE Navigator trends in the reporting of gene variants and odds ratios in PubMed abstracts that were published from 2001 through 2007 (N = 27,132). Overall, 6.3% of abstracts reported rs numbers; 27% reported odds ratios. The proportion of abstracts reporting rs numbers increased substantially (from 1% to 17%) during this period, while the proportion reporting odds ratios remained fairly steady (Fig. 1). Abstracts for genome-wide association studies were more likely than other genetic association studies to include rs numbers (42%) and odds ratios (40%). Conversely, we selected a random 2% sample of all of the extracted PubMed abstracts for hand searching and found that almost all (91%) included common or historical genetic variant names. Matching these common names to the corresponding rs numbers would greatly aid in retrieval and synthesis of genetic association data.

**Figure 2:**
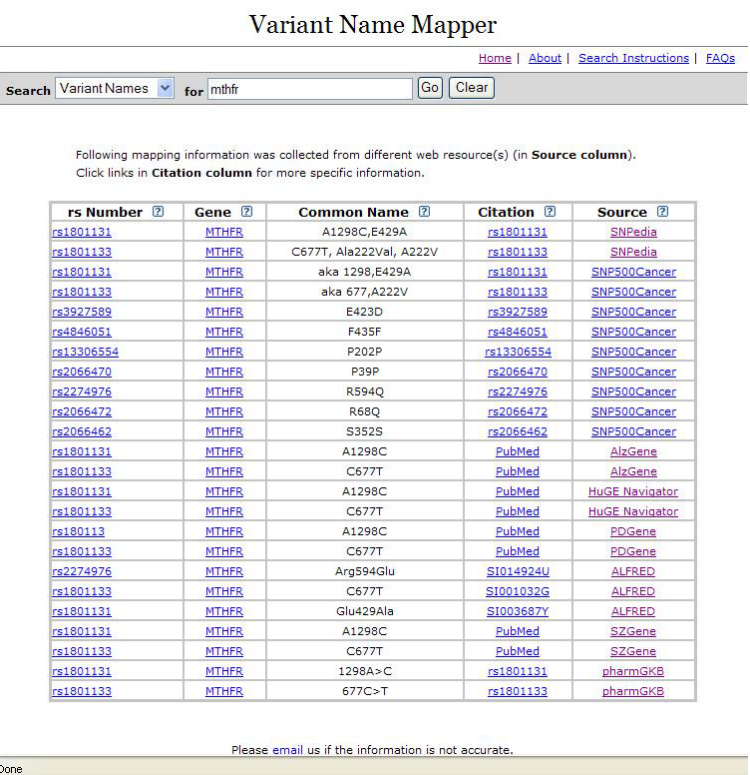
**A screenshot of the Variant Name Mapper**.

To facilitate the mapping of historical names for genetic variants to their rs numbers, we developed a searchable, web-based database called Variant Name Mapper [8]. This database contains historical names matched with their corresponding rs numbers. These data have been extracted from multiple open-access databases, including: SNP500Cancer [9], SNPedia [10], pharmGKB [11], ALFRED [12], AlzGene [13], PDGene [14], SZgene [15], and LSDBs [16], as well as from our own curated data from the HuGE Navigator. User submissions are also welcome. In the Variant Name Mapper, the user is able to search by historical (common) name of the polymorphism, by rs number, or by gene information (including gene symbol, gene name, and gene alias). The display information includes rs number, common/historical polymorphism names, gene-centered information, and a listing of the data sources [Figure 2]. We evaluated the tool's mapping capacity by entering the common names for genetic variants included in the 2% sample of abstracts described above. Overall, 62% of common names could be mapped to an rs number by using the Variant Name Mapper. This low return may be due to the heterogenous nature of the common names and limitations of the data sources. The content of the database will be continually improved and expanded as new data sources become available.

Genome-wide bioinformatics tools, such as HapMap [17] and the UCSC Genome Browser [18], are most useful to researchers for mining genomic information when data can be linked at the variant level. The Human Genome Variation Society (HGVS) has proposed a comprehensive and systematic nomenclature for the description of genetic variants [19]. The combination of dbSNP accession identifiers (rs numbers) with HGVS nomenclature will be beneficial for standardization. The use of standard nomenclatures (e.g., HUGO for genes, dbSNP for gene variants) and systematic reporting of statistics (e.g., odds ratios) in published abstracts would represent an evolutionary advance in information integration and retrieval, which are the first steps in translating genomic research.

## Competing interests

The authors declare that they have no competing interests.

## Authors' contributions

WY drafted the manuscript, and designed and implemented the mapping tool, wrote the source codes. RN was involved in the data extraction and curation and helped in manuscript preparation. AW was involved in the data extraction and the data quality control. TL performed the data preparation and analysis. MJK oversaw the project and revised the draft manuscript. MG provided advice on the project and revised the draft manuscript and led the project. All authors read and approved the final document.
